# Parenchymal‐sparing versus extended hepatectomy for colorectal liver metastases: A systematic review and meta‐analysis

**DOI:** 10.1002/cam4.2515

**Published:** 2019-08-28

**Authors:** Gang Deng, Hui Li, Gui‐qing Jia, Dan Fang, You‐yin Tang, Jie Xie, Ke‐fei Chen, Zhe‐yu Chen

**Affiliations:** ^1^ Department of Liver Surgery and Liver Transplantation Center West China Hospital of Sichuan University Chengdu China; ^2^ Department of Breast Surgery Affiliated Hospital of Guizhou Medical University Guiyang China

**Keywords:** anatomical hepatectomy, colorectal liver metastasis, meta‐analysis, oncological outcomes, parenchymal‐sparing hepatectomy

## Abstract

**Aims:**

To assess the safety and efficacy of parenchymal‐sparing hepatectomy (PSH) as a treatment of colorectal liver metastases (CLM).

**Methods:**

A comprehensive medical literature search was performed. Perioperative and long‐term survival outcomes were pooled. Subgroup analysis and meta‐regression analysis were performed to identify potential sources of heterogeneity.

**Results:**

A total of 18 studies comprising 7081 CLM patients were eligible for this study. The PSH was performed on 3974 (56.1%) patients. We found that the OS (overall survival; hazard ratio [HR] = 1.01, 95% confidence interval [CI]: 0.94‐1.08) and RFS (recurrence‐free survival; HR = 1.00, 95% CI: 0.94‐1.07) were comparable between non‐PSH and PSH group. The perioperative outcomes were better in PSH than in non‐PSH group. Non‐PSH group was significantly associated with longer operative time (standard mean difference [SMD] = 1.17, 95% CI: 0.33‐2.00), increased estimated blood loss (SMD = 1.36, 95% CI: 0.64‐2.07), higher intraoperative transfusion rate (risk ratio [RR] = 2.27, 95% CI: 1.60‐3.23), and more postoperative complications (RR = 1.39, 95% CI: 1.16‐1.66). Meta‐regression analyses revealed that no variable influenced the association between surgical types and the survival outcomes.

**Conclusions:**

This study shows that PSH is associated with better perioperative outcomes without compromising oncological outcomes. Given the increasing incidence of hepatic parenchyma, the PSH treatment offers a greater opportunity of repeat resection for intrahepatic recurrent tumors. It should be considered as an effective surgical approach for CLM.

## Graphical abstract

The present study pooled outcomes of 18 studies to evaluate the safety and effectiveness of parenchymal‐sparing hepatectomy (PSH) for treating colorectal liver metastases (CLM). PSH is associated with potential better perioperative outcomes without compromising oncological outcomes. More future hepatic parenchymal will be preserved in the surgical process of PSH, it offers a greater opportunity of repeat resection for intrahepatic recurrent tumors. The PSH brings the treatment of CLM into personalized precision medicine era.

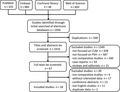

## 1 INTRODUCTION

Colorectal cancer (CRC) is the third most commonly diagnosed cancer which accounts for approximately 9.2% of cancer‐related deaths.^1^ A large proportion of patients with CRC present with synchronous liver metastases either during the initial stages or at the advanced stages.^2^ Colorectal liver metastasis (CLM) is the main cause of tumor‐related death in patients with CRC.^2^, ^3^ During the past two decades, several treatment strategies have been developed for CLM. Curative liver resection results in better outcomes than radiofrequency ablation or chemotherapies alone when carefully applied to patients with resectable metastases.^4^, ^5^ However, despite the advances in surgical skills and multidisciplinary treatment strategies, only 25% patients with CLM are eligible for this operation.^6^ In addition, more than half of patients will develop early tumor relapse in the remnant liver within two postoperative years.^7^ Thus, treatments for CLM should not only cure the tumor, but also prevent tumor recurrence and improve outcomes.

Initially, major resection (MR) and anatomical hepatectomy (AH) were used to treat CLM since they offered sufficient resection margin.^8^, ^9^, ^10^ Theoretically, MR and AH are associated with less intrahepatic tumor relapse or metastases due to the extended resection of potentially “tumor‐bearing” portal tributaries.^3^, ^11^ Recently, parenchymal‐sparing hepatectomy (PSH) has become a major treatment for CLM.^12^, ^13^ PSH leaves a more functional remnant liver, is less invasive and produces better short‐term outcomes.^14^, ^15^ Besides, PSH can even be performed for CLM patients with technically challenging tumors with the guidance of intraoperative ultrasound.^12^, ^16^ Several studies have compared outcomes of PSH with those of non‐PSH. Findings from such studies have been varied with respect to the application of PSH for CLM.

The primary aim of the present study is to compare the perioperative and long‐term oncological outcomes between PSH and non‐PSH. Moreover, subgroup analysis was conducted according to specific surgical types and geographical regions.

## 2 METHODS

### 2.1 Literature search strategy and inclusion/exclusion criteria

The present meta‐analysis was performed according to the Preferred Reporting Items for Systematic Reviews and Meta‐Analyses (PRISMA) guidelines.^17^ Medical databases PubMed, Embase, Cochrane Library, and Web of Science were comprehensively searched in September 2018 to identify eligible studies on the types of resection for CLM. The literature search was performed without restriction on publication language, types, or geographical regions. Search terms were: (colorectal OR rectal OR colon) AND (liver OR hepatic) AND resection AND (metastasis OR metastases) AND (parenchyma OR parenchymal). Two authors completed the literature screening independently by reading titles and abstracts of eligible studies. In addition, a backward scrutinization was performed by cross‐checking the reference lists of review articles and other studies.

The PSH reported in this study comprised of wedge resection, minor hepatectomy with parenchymal‐sparing approach and non‐anatomical metastasectomy. For studies that compared segmental resection to extended hepatectomy, we defined segmental resections as PSH since they preserved significant liver parenchyma.^13^, ^18^, ^19^

Studies which met the following criteria were included: studies that evaluated the technical and oncologic feasibility of PSH for CLM; the types of resection were considered as variables in outcome analysis; studies that compared PSH and non‐PSH techniques for liver resection; studies that provided data on the long‐term survival outcomes or perioperative outcomes.

The criteria for exclusion were as follows: studies that did not focus on PSH; those that did not compare the two treatments; review articles and editorials; animal researches; conference abstracts or case reports; studies without perioperative or survival outcomes.

**Figure 1:**
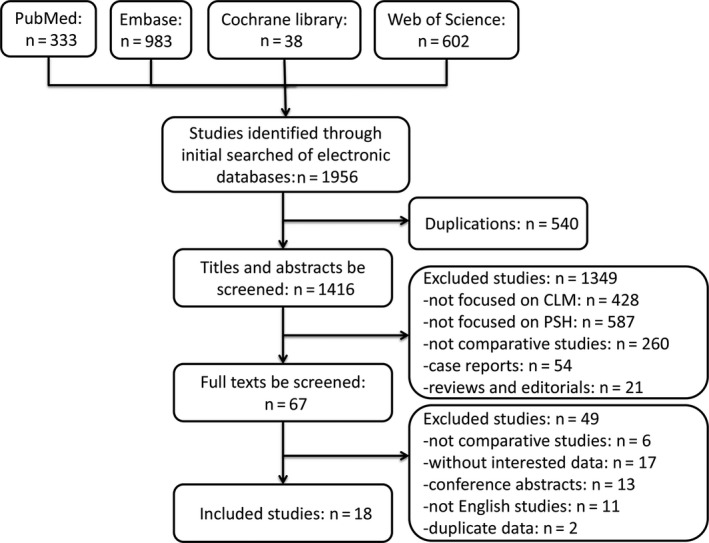
Flow diagram showing study retrieval and selection process

For overlapping publications by same authors or centers, only those with largest patient cohorts were included in the present meta‐analysis.^20^ For studies that analyzed two or more independent sample sets, such as experiment and validation cohorts, both were included and analyzed independently. Figure 1 shows the detailed search strategy.

### 2.2 Data management

The software EndNote (version X8) was used to sort and manage the preliminarily selected studies. Two authors performed data extraction independently. Any discrepancy observed was settled by team consensus. After reading the full text of included studies, the raw data were extracted and summarized in tables. The long‐term survival and oncological outcomes were primary outcomes of this meta‐analysis, which included overall survival (OS) and recurrence‐free survival (RFS). Secondary outcomes were perioperative data included operative time, estimated blood loss (EBL), intraoperative blood transfusion, length of hospitalization (LOH), postoperative complications, positive margin rate, 30‐day mortality and 90‐day mortality. The survival outcomes were compared by pooling hazard ratio (HR) and 95% confidence interval (CIs). Data on outcomes of univariable or multivariable analysis were extracted from tables or texts. For outcomes that were not summarized directly, the HRs and 95% CIs were calculated from the Kaplan‐Meier curves using the Engauge Digitizer software (Version 4.1).^21^

### 2.3 Quality assessment and statistical analysis

The modified Newcastle‐Ottawa scale was used to evaluate the methodological quality of all included cohort studies.^22^, ^23^ The assessment comprised of three sections: patient selection, comparability and assessment of outcomes. The maximum total score was 9. The detailed lists of grading rules are shown in Table S1. A follow‐up period of at least 2 years was considered adequate. A maximum rate of loss to follow‐up of less than 20% was considered acceptable. Studies that achieved a score of ≥ 6 were considered as high quality.

Results were pooled using the Cochrane Collaboration's Review Manager software (version 5.3, Cochrane Collaboration). Statistical heterogeneity among studies was explored using the χ2 test and was considered significant at a *P*‐value of .10. Heterogeneity was quantified using I^2^ statistic. The fixed effects model was used if there was no obvious heterogeneity among the enrolled studies (*I* ^2^ < 40), otherwise random effects model was used.^23^

### 2.4 Sensitivity analysis and publication bias

Sensitivity analysis was performed using Stata (version 12.0, Stata Corp), by omitting enrolled studies, one at a time, to investigate the stability of the synthesized outcomes. Subsequently, the results were pooled afresh after excluding studies with low‐quality (less than 6 points).

The funnel plot was used to investigate the publication bias. The symmetry of the funnel plot was examined by Egger and Begg tests (Stata version 12.0).^24^

### 2.5 Subgroup analysis and meta‐regression

Subgroup analyses were performed according to the surgical types of non‐PSH (such as anatomic liver resection, hemihepatectomy, and major hepatectomy).

Meta‐regression analysis was carried out using Stata (version 12.0), to evaluate the potential influence of various variables on the association between surgical types and survival outcomes. Meta‐regression analysis was only performed for OS and RFS due to sufficient study arms.

## 3 RESULTS

### 3.1 Characteristics of the included studies

As shown in Figure 1, a total of 18 studies were included in the present meta‐analysis after rigorous screening.^3^, ^13^, ^18^, ^19^, ^25^, ^26^, ^27^, ^28^, ^29^, ^30^, ^31^, ^32^, ^33^, ^34^, ^35^, ^36^, ^37^, ^38^ After excluding duplicate studies, a total of 1416 studies were screened by reading the abstracts. The characteristics of included studies are summarized in Table 1. A total of 7081 patients with CLM who underwent liver resection from January 1980 to December 2015 were included. Among them, 3974 (56.1%) underwent PSH, whereas 3107 (43.9%) received non‐PSH. The methodological quality of cohorts and case‐control studies were presented as five‐pointed stars. Although the study by Inoue et al^34^ was a brief report, it was included because it provided data of OS and RFS. The studies by Matsuki et al^39^ and Raoof et al^40^ overlapped with those of Matsumura et al^32^ and Mise et al^31^ All studies were of high‐quality evidence except a 5‐point study^13^ (Table S1).

**Table 1:** Characteristics of eligible studies

References	Study period	Country	Design/Centers	Patients		Male (%)	Age	Location of the primary site	Synchronous liver metastases (%)	Number of metastases	Largest metastasis size	Operative procedures		Negative margin (%)	Follow up	Type of survival outcomes	Adjuvant therapy	Quality
				PSH	Non‐PSH							PSH group	NPSH group					
DeMatteo et al^35^	1985.6‐1998.10	USA	R/Single	119	148	155 (58)	65 (28‐87)	C: 196; R: 71	60 (22)	Single: 214; multiple: 53	3 (0.4‐19)	Wedge resection	Segmental resection	245 (92)	25 (1‐140)	1, 3, 5 year‐OS; 1 year‐DFS	AC: 22	★★★★★★★
Donadon et al^38^	2001.2‐2013.6	Italy	R/2 centers	110	110	141 (64)	62.9 ± 10.6	N/A	87 (40)	3.6 ± 3.0	4.7 ± 2.5	Minor hepatectomy with parenchymal‐sparing approach	Remove at least 3 adjacent segments	184 (84)	33 (1‐83)	1, 3, 5 year‐OS; 1, 3, 5 year‐DFS	AC: 156; NAC: 129	★★★★★★
Finch et al^37^	1993.1‐2003.5	UK	P/Single	96	280	235 (63)	63 (24‐84)	N/A	153 (41)	2 (1‐14)	4 (0.4‐20)	Metastasectomy	Anatomical hepatectomy	101 (73)	33 (24‐144)	OS; DFS	AC: 376	★★★★★★
Guzzetti et al^36^	1996.12‐2005.12	Italy	Rp/Single	106	102	128 (58)	<70:155;>= 70:53	C: 115; R: 52	N/A	Single: 126; multiple: 74	<5:145;>=5:52	Wedge resection	Anatomical resection	159 (76)	N/A	1, 3, 5 year‐OS; 1 year‐DFS	AC: 115	★★★★★★
Hosokawa et al^26^	2000.1‐2015.12	Japan	R/Five	1478	242	1029 (60)	64.1 (10.9)	C: 1138; R: 525	842 (49)	N/A	1.9 (0.8)	Parenchyma‐preserving hepatectomy	Right hepatectomy	1453 (84)	Mean: 41	OS; RFS	AC: 852; NAC: 539	★★★★★★
Inoue et al^34^	2001.4‐2015.12	Japan	R/Single	215	57	N/A	N/A	N/A	N/A	N/A	N/A	Parenchymal‐sparing hepatectomy	Major hepatectomy	N/A	N/A	OS; RFS	N/A	Brief report
Kokudo et al^25^	1980.1‐1999.12	Japan	R/Single	78	96	100 (57.5)	59.4 ± 1.4	C: 120; R: 54	102 (58.6)	Single: 96; multiple: 78	4.4 ± 1.6	Limited wedge resection	Major hepatic resection	132 (76.9)	N/A	OS	N/A	★★★★★★★
Lalmahomed et al^33^	2000.1‐2008.6	Netherland	R/Single	113	88	126 (63)	65 (30‐86)	C: 114; R: 87	78 (39)	1 (1‐8)	3 (0.5‐15)	Wedge hepatectomy	Remove at least 2 segments or hemihepatectomy	181 (90)	35 (1‐111)	OS; RFS	NAC: 59	★★★★★★★★
Lordan et al^18^	2000.1‐2010.12	UK	R/Single	238	238	265 (55.7)	65.3 (24‐87)	N/A	29 (6.1)	Single: 314; multiple: 162	3.15 ± 1.8	Remove less than 3 segments	Remove at least 3 segments	431 (90.5)	36 (0.12‐144)	OS; RFS	AC: 274; NAC: 37	★★★★★★★
Margonis et al^41^	2000.1‐2015.6	USA	R/Single	165	224	231 (59.4)	58.4 (50.1‐66.4)	C: 302; R: 87	223 (57.3)	2 (1‐3)	2.5 (1.3‐3.0)	Wedge hepatectomy	Remove at least 2 segments	302 (77.6)	Median: 28	RFS	AC: 256	★★★★★★★
Matsumura et al^32^	1999.1‐2012.12	Japan	RP/Single	113	32	96 (66.2)	60 (27‐81)	C: 87; R: 58	N/A	6 (4‐33)	2.5 (0.4‐5)	Parenchyma‐preserving hepatectomy	Major hepatectomy	132 (91)	N/A	OS; RFS	NAC: 100	★★★★★★
Memeo et al^19^	2006.1‐2013.12	France	R/32 centers	331	360	385 (55.7)	61 (27‐82)	C: 524; R: 167	294 (42.5)	4.5 (3‐14)	3 (0.4‐3)	Maximum of 1 segment resection	At least 3 consecutive segments resection	482 (69.8)	N/A	OS; RFS	AC: 390; NAC: 237	★★★★★★
Mise et al^31^	1993.1‐2013.12	USA	RP/Single	156	144	174 (58)	60 (22‐88)	C: 218; R: 82	N/A	N/A	1.5 (0.3‐3)	Partial wedge hepatectomy	Anatomic hepatectomy	293 (97.7)	37 (2‐208)	OS; RFS	NAC: 170	★★★★★★
Pandanaboyana et al^30^	1993.1‐2011.8	UK	RP/Single	409	582	N/A	66 (IQR 23.8‐91.8)	N/A	522 (52.7)	2 (IQR 1‐3)	N/A	Metastatectomy	Remove at least 2 segments	735 (74.2)	33.2 (IQR 17.5‐56.9)	OS; RFS	NAC: 410	★★★★★★
Sarpel et al^29^	1987.8‐2007.8	USA	R/Single	89	94	105 (57)	62 (31‐90)	C: 22; R: 119	N/A	1.5 (1‐7)	5.1 (0.5‐24.5)	Wedge resection	Anatomical resection	155 (84.7)	Median: 34	Mean survival	N/A	★★★★★★
Spelt et al^28^	2006.1‐2014.12	Sweden	R/Single	59	60	74 (62)	N/A	N/A	67 (56.3)	N/A	N/A	Wedge resection	At least hemihepatectomy	N/A	Median: 35	OS	NAC: 87	★★★★★★
Stewart et al^13^	1988.10‐2001.4	UK	R/Single	27	69	N/A	Range 28‐82	N/A	N/A	1‐3:87; >3:9	>5:50; <5:28	Wedge or segmental resection	Left or right hepatectomy	75 (96.2)	N/A	OS	N/A	★★★★★
Zorzi et al^27^	1999.3‐2004.5	USA, Italy, Switzerland	R/Multicenters	72	181	159 (63)	61 (32‐88)	C: 155; R: 63	98 (38.8)	Single: 144; multiple: 109	2.7 (0.3‐18)	Wedge resection	Anatomic resection at least 1 segment	232 (91.7)	Median: 25	OS	N/A	★★★★★★

Abbreviations: AC, adjuvant chemotherapy; C, colon; N/A, not available; NAC, neoadjuvant chemotherapy; OS, overall survival; P, prospective; PH, prospectively design historically control; PSH, parenchymal‐sparing hepatectomy; R, rectum; R, retrospective; RCT, randomized controlled trail; RFS, recurrence‐free survival; RP, retrospectively design prospectively collect.

### 3.2 Primary outcomes

**Figure 2:**
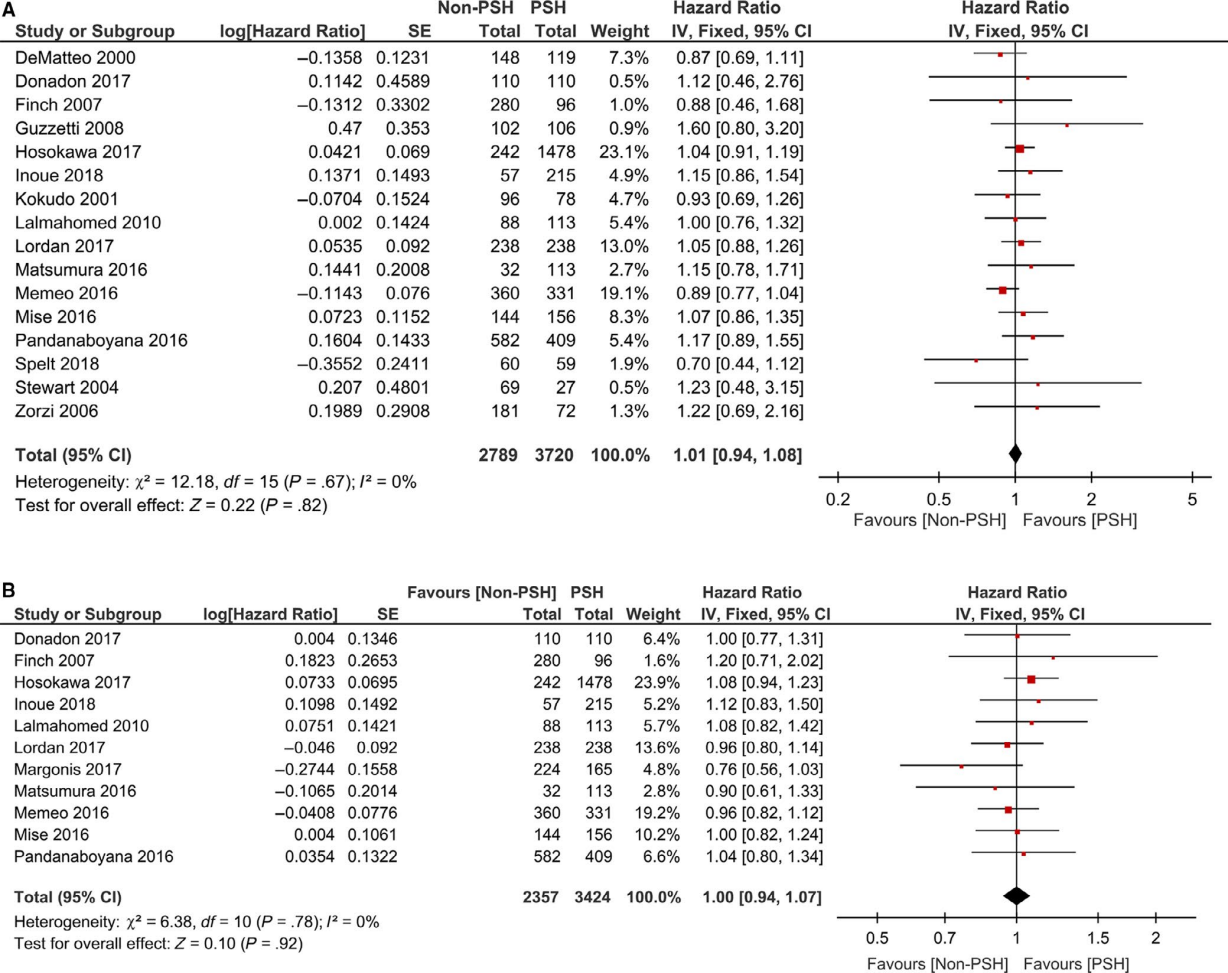
Forest plots showing association between surgical types and overall survival (A) and recurrence‐free survival (B)

The pooled primary outcomes are summarized in Table 2 and Figure 2. Sixteen studies showed that the OS was comparable between non‐PSH and PSH groups (HR = 1.01, 95% CI: 0.94‐1.08, *P* = .82). Eleven studies revealed that the recurrence rate of RFS was similar between PSH and non‐PSH (HR = 1.00, 95% CI: 0.94‐1.07, *P* = .92). Separate analyses demonstrated a comparable 3‐year and 5‐year OS between PSH and non‐PSH (RR = 1.00, 95% CI: 0.91‐1.10 and HR = 0.93, 95% CI: 0.82‐1.05, respectively) (Figure S1). Similarly, separate analyses showed that the 1‐, 3‐, and 5‐year RFS were comparable between PSH and non‐PSH groups (RR = 0.96, 95% CI: 0.89‐1.04, RR = 0.97, 95% CI: 0.90‐1.05 and RR = 1.19, 95% CI: 0.91‐1.56, respectively) (Figure S2).

**Table 2:** Results of meta‐analysis comparison of PSH and non‐PSH

Outcomes of interest	Studies	Patients		SMD/RR/HR (95% CI)	*P* value	Study heterogeneity			
		Non‐PSH	PSH			x^2^	*df*	I^2^,%	*P* value
Long‐term outcomes									
Overall survival	16	2789	3720	1.01 (0.94, 1.08)	.82	12.18	15	0	.67
1‐year OS	7	1235	1054	0.95 (0.90, 1.00)	.04	27.88	6	78	<.001
3‐year OS	8	1477	2532	1.00 (0.91, 1.10)	.93	17.43	7	60	.01
5‐year OS	11	1722	2828	0.93 (0.82, 1.05)	.26	25.73	10	61	.004
Recurrence‐free survival	11	2357	3424	1.00 (0.94, 1.07)	.92	6.38	10	0	.78
1‐year RFS	3	708	679	0.96 (0.89, 1.04)	.31	2.69	2	26	.26
3‐year RFS	4	950	2157	0.97 (0.90, 1.05)	.50	2.17	3	0	.54
5‐year RFS	4	950	2157	1.19 (0.91, 1.56)	.21	12.61	3	76	.006
Short‐term outcomes									
Operative time [min]	8	1023	989	1.17 (0.33, 2.00)	.006	460.00	7	98	<.001
Estimated blood loss [mL]	8	916	823	1.36 (0.64, 2.07)	<.001	291.56	7	98	<.001
Intraoperative blood transfusion	7	1653	2709	2.27 (1.60, 3.23)	<.001	18.72	6	68	.005
Length of hospital stay [days]	10	1795	2742	0.10 (0.00, 0.20)	.06	19.60	9	54	.02
Postoperative complications	16	2997	3670	1.39 (1.16, 1.66)	<.001	48.29	15	69	<.001
Positive margin	15	2929	3579	0.86 (0.71, 1.03)	.09	25.75	14	46	.03
30‐day mortality	7	827	608	1.54 (0.63, 3.76)	.34	3.11	6	0	.79
90‐day mortality	6	1466	1357	3.36 (1.71, 6.60)	<.001	2.96	4	0	.56

Abbreviations: CI, confidence interval; *df*, degrees of freedom; HR, hazard ratio; NA, not available; OR, odds ratio; PSH, parenchymal‐sparing hepatectomy; RR, risk ratio; SMD, standard mean difference.

### 3.3 Perioperative outcomes

Eight studies with 2012 patients demonstrated that the non‐PSH needed more operative time (standard mean difference [SMD] = 1.17, 95% CI: 0.33‐2.00, *P* = .006) compared to PSH (Table 2). Non‐PSH was associated with increased EBL (SMD = 1.36, 95% CI: 0.64‐2.07) and higher intraoperative transfusion rate (RR = 2.27, 95% CI: 1.60‐3.23) compared to PSH. LOH and positive margin rate were comparable between PSH and non‐PSH groups. Importantly, non‐PSH was associated with more postoperative complications than PSH (RR = 1.39, 95% CI: 1.16‐1.66). The 90 days mortality rate was higher in non‐PSH than in PSH (RR = 3.36, 95% CI: 1.71‐6.60) (Figure S3).

### 3.4 Sensitivity analysis and publication bias

**Figure 3:**
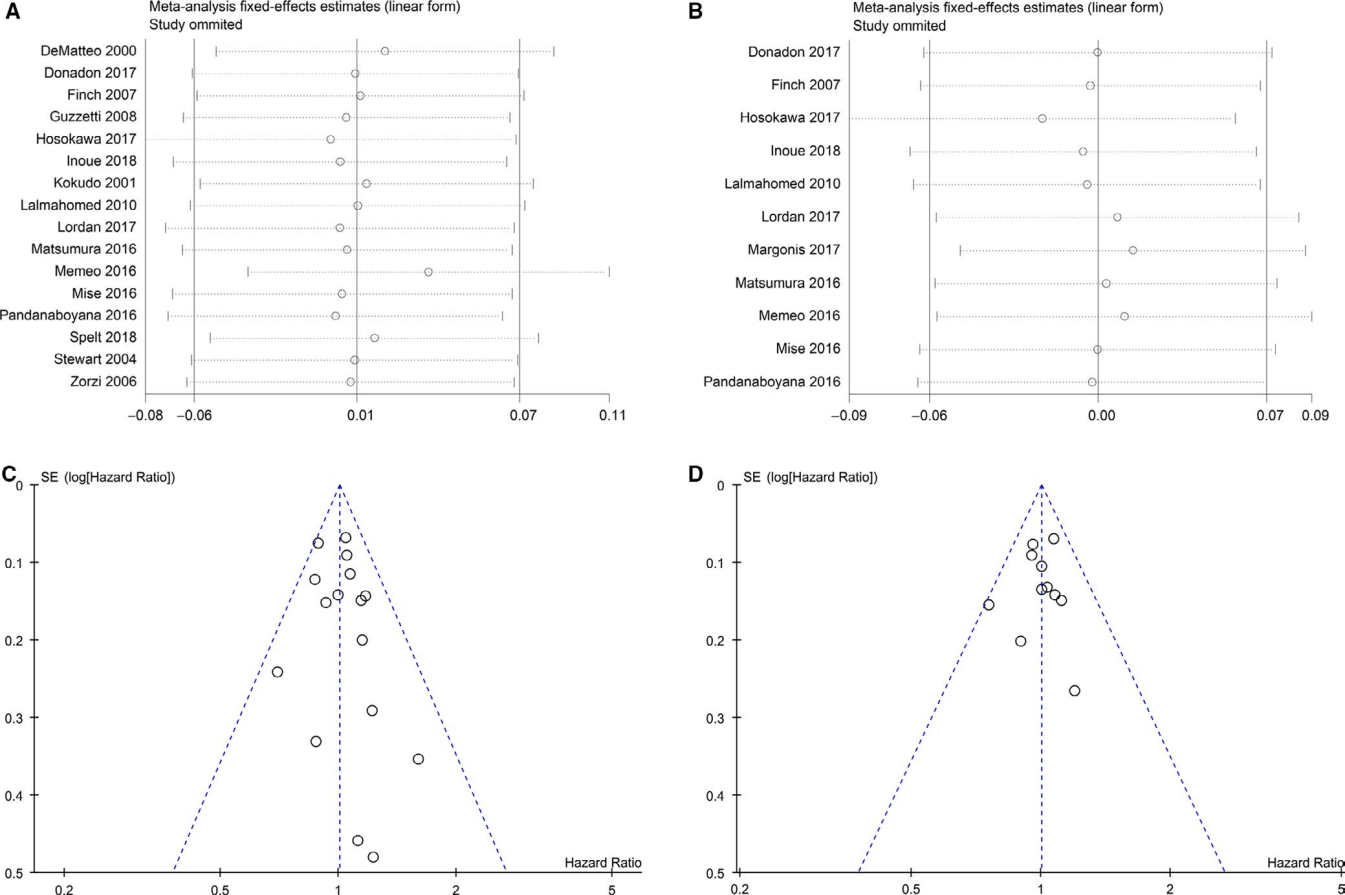
Sensitivity analyses and funnel plots of overall survival (A, C) and recurrence‐free survival (B, D)

A sensitivity analysis was performed by omitting the included studies one at a time to investigate the stability of the obtained OS and RFS. As shown in Figure 3, the pooled HRs were not significantly altered after eliminating the included studies in turns.

The funnel plot was used to show publication bias of OS and RFS (Figure 3). The studies were almost symmetrically distributed around the central line and were inside the 95% CIs. Besides, two statistical tests were applied to evaluate the dissymmetry of the funnel plots: the Begg (z = 0.59, *P* = .558) and Egger (bias coefficient 0.412, standard error 0.465, *t* = 0.89, *P* = .391). These outcomes demonstrated that there was no significant publication bias among the included studies.

### 3.5 Subgroup analysis and meta‐regression analysis

The results of the subgroup analyses are summarized in Table 3. Compared to AH, non‐PSH produced favorable perioperative or survival outcomes for CLM patients. Consistent with our overall results, AH was associated with increased operative time and EBL (pooled SMD = 1.50, and 1.42, respectively). The postoperative complication rate was similar between AH and PSH (RR = 1.28, 95% CI: 0.95, 1.71). The OS was comparable between AH and PSH (HR = 1.02, 95% CI: 0.91, 1.14). Similarly, there was no significant difference in RFS between AH and PSH (pooled HR = 0.99, 95% CI: 0.87, 1.12).

**Table 3:** Subgroup analysis of anatomic versus non‐anatomic hepatectomy

Outcomes of interest	Studies	Patients		SMD/RR/HR	*P* value	Study heterogeneity			
		Non‐PSH	PSH	(95% CI)		x^2^	*df*	I^2^,%	*P* value
Long‐term outcomes									
Overall survival	8	1621	1149	1.02 (0.91, 1.14)	.72	5.35	7	0	.62
Recurrence‐free survival	5	1318	939	0.99 (0.87, 1.12)	.87	3.90	4	0	.42
Short‐term outcomes									
Operative time [min]	4	461	459	1.50 (0.12, 2.88)	.03	255.34	3	99	<.001
Estimated blood loss [mL]	5	714	624	1.42 (0.56, 2.28)	.001	202.05	4	98	<.001
Intraoperative blood transfusion	2	671	522	2.99 (0.97, 9.28)	.06	7.58	1	87	.006
Length of hospital stay [days]	5	735	558	0.06 (−0.13, 0.25)	.56	11.37	4	65	.02
Postoperative complications	9	1845	1314	1.28 (0.95, 1.71)	.11	31.07	8	74	<.001
Positive margin	9	1851	1290	0.85 (0.63, 1.14)	.29	21.80	8	63	.005
30‐day mortality	5	607	471	1.71 (0.58, 5.04)	.33	2.66	4	0	.62
90‐day mortality	2	726	565	4.01 (1.63, 9.90)	.003	0.02	1	0	.89

Abbreviations: CI, confidence interval; *df*, degrees of freedom; HR, hazard ratio; NA, not available; OR, odds ratio; PSH, parenchymal‐sparing hepatectomy; RR, risk ratio; SMD, standard mean difference.

Meta‐regression analysis was performed to determine the influence of publication year, percentage of males, age, location of primary tumors (percentage of colon cancer), number of metastases, size of largest metastases, percentage of negative margin, follow‐up period, percentage of neoadjuvant chemotherapy and quality scores, on the association between surgical types with OS and RFS. (Table S2).

## 4 DISCUSSION

In the present meta‐analysis, we found that PSH was associated with better perioperative outcomes compared with non‐PSH. Importantly, the OS and RFS were favorable in PSH. In terms of surgical margin, PSH and non‐PSH had comparable positive margin rate. One of the key targets of CLM resection is to achieve a successful R0 resection with sufficient surgical margin. An R0 resection of metastases with a surgical margin of >1 cm was associated with better oncological outcomes in patients with CLM.^41^ Our findings based on 7081 patients show that the positive margin rate is similar between PSH and non‐PSH. Indeed, with the application of intraoperative ultrasound, it is easier to identify metastases as well as the relationship between lesions in the vascular structures, which facilitates the achievement of an R0 surgical margin.^16^, ^17^

A previous meta‐analysis by Sui et al compared anatomic with nonanatomic liver resection for CLM.^42^ They included seven studies with 1662 patients and demonstrated that nonanatomic resection did not compromise oncological outcomes of CLM patients. Moris and colleagues performed a systematic review to compare PSH with AH. They revealed that PSH was associated with a comparable safety and efficacy profile compared with AR and did not compromise oncologic outcomes.^43^ However, non‐PSH not only contains anatomical resection. If a right liver is affected by several metastases, an anatomical right hemihepatectomy may be considered as PSH although it is a major liver resection.^44^ A meta‐analysis by Tang et al also compared the perioperative and survival outcomes of anatomic versus nonanatomic hepatectomy, but their inclusion criteria and assessment of methodological quality were not sufficiently rigorous.^45^ Moreover, they included studies without comparable data in their study.^46^ In the present meta‐analysis, we included many studies comparing segmental resection with extended hepatectomy.^13^, ^18^, ^19^ We considered the segmental resections as PSH since they preserved sufficient liver parenchyma during resection. Nevertheless, there is no standard definition for PSH. In clinical practice, to perform PSH for CLM patients, collaboration across expert networks involving surgeons, pathologists and oncologists is needed, especially for advanced tumors with multiple metastases.^44^ The incorporation of preoperative images with intraoperative findings will facilitate more accurate resection of metastases. Postoperative pathological evaluation provides an integrated review of surgical procedures and assessment of clinical outcomes. A combination of these approaches may produce more accurate estimates of tumor volume and definition of PSH.

The main concern regarding the use of PSH is whether it will increase the risk of positive surgical margins. In the present analysis, the pooled outcome of 15 studies demonstrated that the incidence of positive margin was comparable between PSH and non‐PSH. A recent meta‐analysis by Margonis et al suggested that a >1 mm surgical margin was associated with better prognostic outcome than a submillimeter surgical margin for CLM.^41^ In contrast, several studies reported similar prognostic outcome for surgical margins with 1‐9.9 mm and >1cm for R0 resection.^47^, ^48^, ^49^ The occurrence of micrometastases is mainly restricted to the area adjacent to the tumor border, yet rare in hepatic parenchyma surrounding CLM.^42^, ^50^ Hepatectomy is recommended for CLM because it allows complete macroscopic removal of metastases with R0 resection irrespective of the width of surgical margin.^51^ Compared to surgical approaches, recent studies have demonstrated that tumor biology can predict oncological outcomes of CLM patients. Margonis et al compared the benefits of AH versus non‐AH in 389 CLM patients. They found that 140 patients had KRAS mutation.^3^ In their study, DFS was comparable between R0 resection with different widths in KRAS wide‐type cancers, whereas nonanatomic hepatectomies were associated with earlier tumor relapse in patients with KRAS‐mutated CLM.

In non‐PSH, portal vein occlusion is more frequently performed compared to PSH due to the extended removal of liver parenchyma. Cytokines such as transforming growth factor‐β and interleukins, which regulate hepatocyte homoeostasis as well as residual micrometastases growth, are more likely to release.^52^, ^53^ In addition, Zhang et al demonstrated that removal of larger volume of liver parenchyma would promote tumor growth in vivo by activating Hedgehog signaling.^54^ Theoretically, incorporation of surgical procedures like hepatic pedicle dissection to non‐PSH prolongs the operative time. Our results confirm that PSH is associated with more operative time than non‐PSH. Furthermore, the pooled outcomes showed that estimated operative blood loss and intraoperative transfusion were lower in PSH than in non‐PSH. In PSH, a smaller volume of liver parenchyma is removed, resulting in low blood loss. However, in non‐PSH, especially anatomic hepatectomy, the presence of an “avascular” intersegmental plane would be transected, resulting in mitigated blood loss per transected area.^3^ Therefore, further well‐designed studies are needed to verify our findings.

Additionally, our results demonstrate that PSH is associated with fewer postoperative complications than non‐PSH. A longer operative duration and higher hepatic parenchyma loss will increase the risk of postoperative infection, ascites, and liver failure. Since PSH aims at removing the metastases as well as minimizing resection of normal hepatic parenchyma, it is not surprising that the incidence of liver insufficiency is frequent in non‐PSH. Subgroup analyses according to specific postoperative complications were not performed owing to insufficient data.

However, there are several limitations that should be considered when interpreting our findings. Firstly, no random controlled study was included in the present study. Secondly, selection biases were inevitable in several retrospective studies. Patients with larger tumors or vascular invasion are more likely to undergo extended hepatectomy, whereas patients with worse preoperative liver function or smaller tumors tended to receive PSH. Future well‐designed random controlled studies are needed to validate the stability of our findings. Furthermore, differences in surgical skills and experience may affect perioperative outcomes particularly EBL, operative time and transection time. Moreover, the management policies for patients were not uniform across institutions, resulting in heterogeneities in LOH. Finally, some data were not included in databases. Nevertheless, this is the most comprehensive meta‐analysis evaluating the effectiveness of PSH in the largest sample of CLM patients.

In conclusion, this meta‐analysis suggests that PSH is safer and more effective for patients with CLM compared to extended hepatectomy. PSH is associated with favorable perioperative outcomes without compromising oncological outcomes. Given that PSH preserves more hepatic parenchyma, it allows for repeat resection of intrahepatic recurrent tumors. It can therefore be considered that PSH treatment for CLM promotes personalized precision medicine and is an appropriate surgical approach for patients with CLM.

## CONFLICT OF INTEREST

None declared.
