# Supplementary figures and images for: Parenchymal‐sparing versus extended hepatectomy for colorectal liver metastases: A systematic review and meta‐analysis
Source: Cancer Med. 2019 Aug 28;8(14):6165–75. doi: 10.1002/cam4.2515 (PMC6797569; doi:10.1002/cam4.2515)

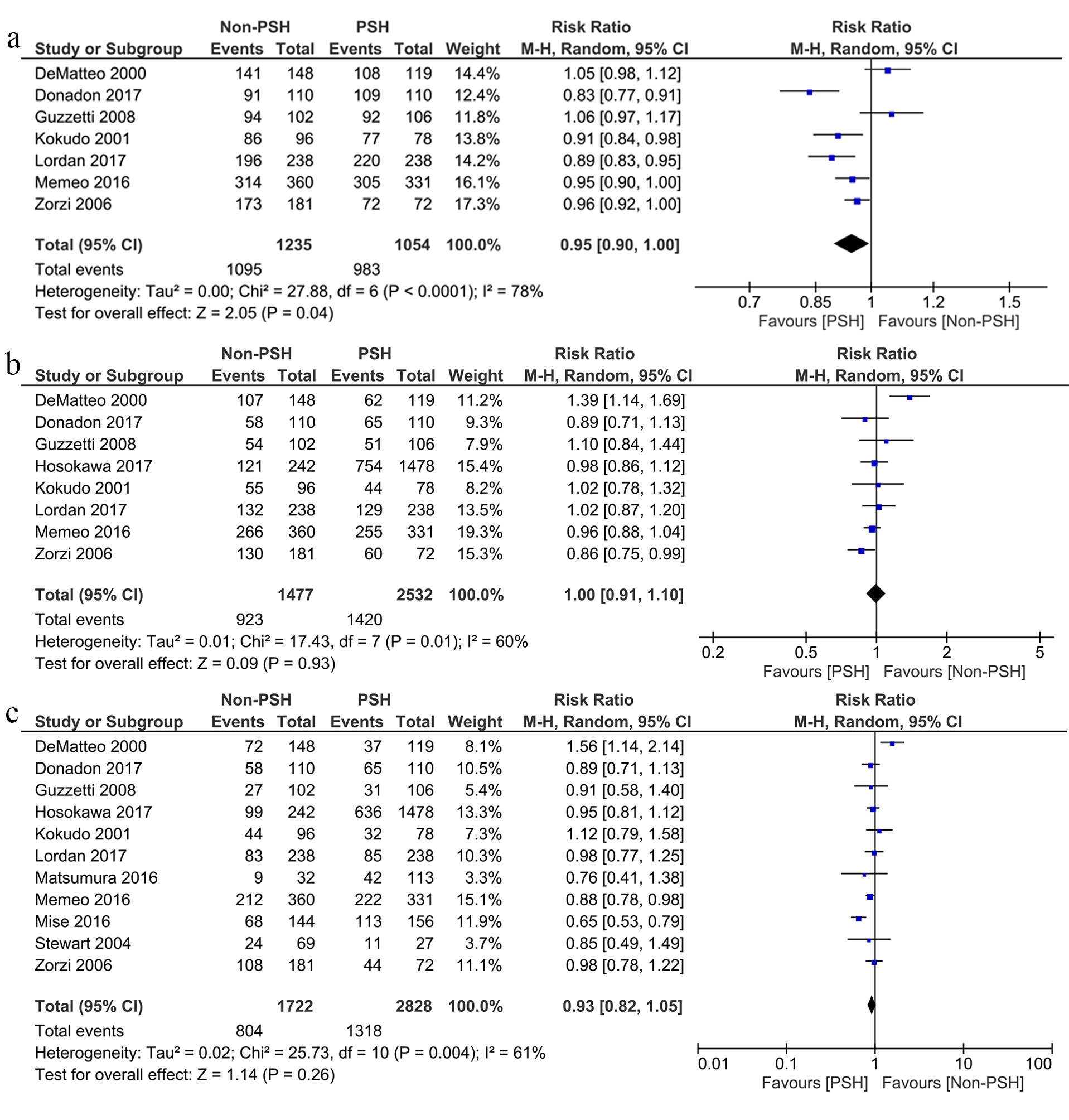

Supplement: Supplementary file 1 [file CAM4-8-6165-s001.tif]

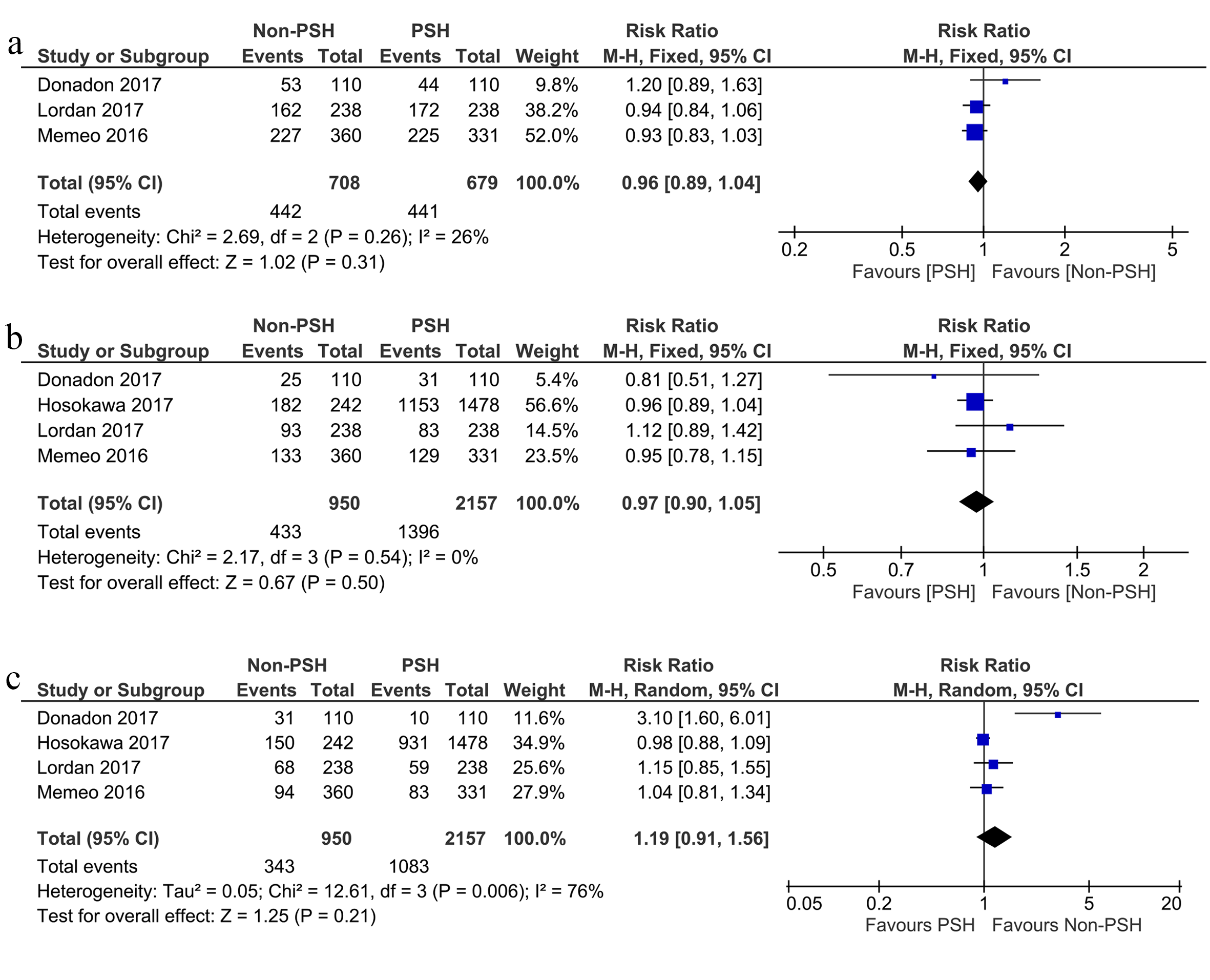

Supplement: Supplementary file 2 [file CAM4-8-6165-s002.tif]

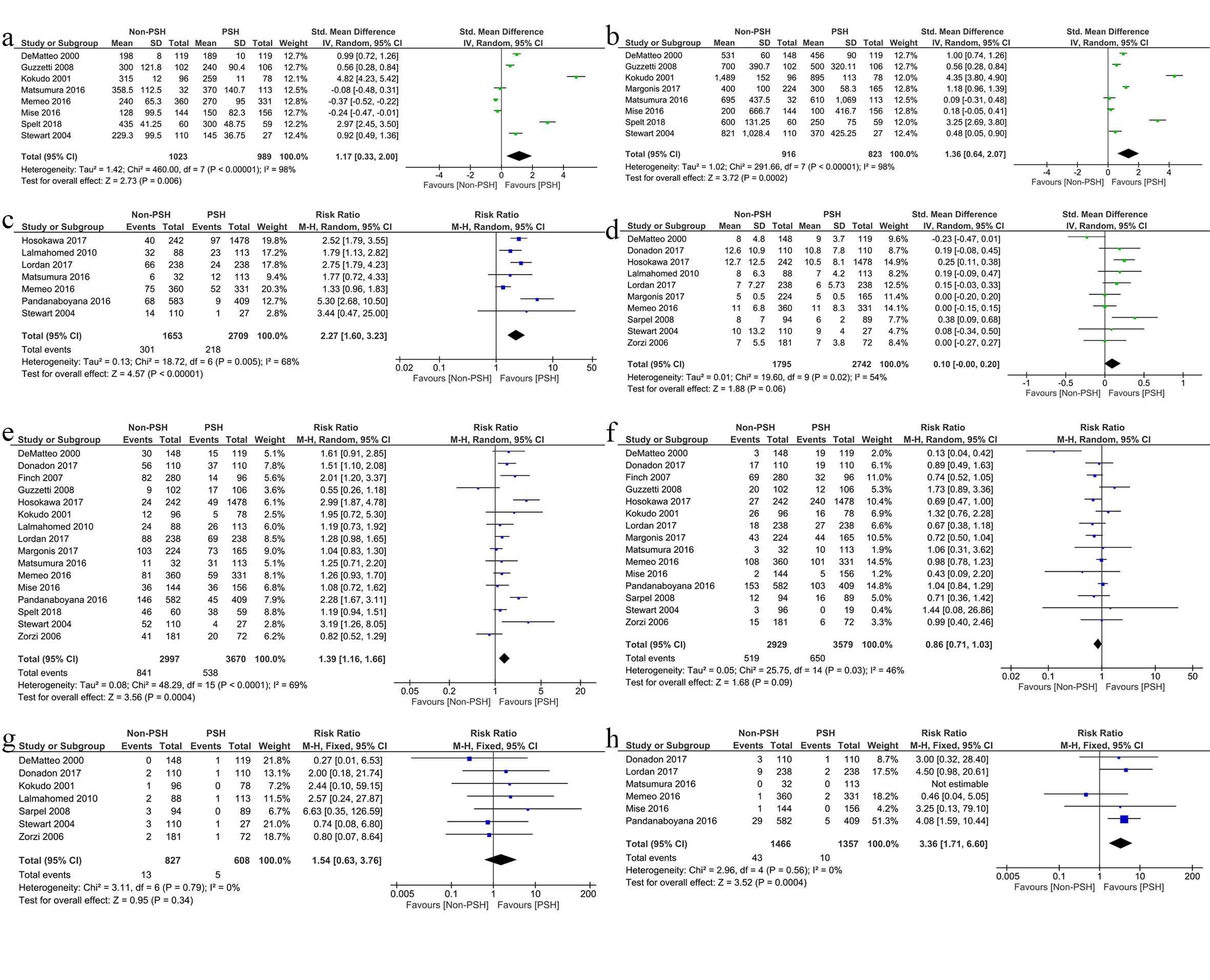

Supplement: Supplementary file 3 [file CAM4-8-6165-s003.tif]
