# Supplementary material for: Parenchymal‐sparing versus extended hepatectomy for colorectal liver metastases: A systematic review and meta‐analysis
Source: Cancer Med. 2019 Aug 28;8(14):6165–75. doi: 10.1002/cam4.2515 (PMC6797569; doi:10.1002/cam4.2515)
Supplement: Supplementary file 4 [file CAM4-8-6165-s004.docx]

**Supplemental table 1.** Qualities of cohort studies evaluated by modified Newcastle-Ottawa scale.

|  |  | selection | |  | Comparability* | | Outcomes | |  |
| --- | --- | --- | --- | --- | --- | --- | --- | --- | --- |
| Studies | Case definition | Representativeness | Selection of controls | Definition of controls | Comparable for 1, 2, 3, 4, 5† | Comparable for 6, 7, 8, 9, 10 | Assessment of outcomes | Integrity of follow-up | Quality score |
| DeMatteo et al. | Yes | Yes | No | Yes | 2, 4, 5 | 6, 7, 8, 10 | Yes | Yes | ★★★★★★★ |
| Donadon et al. | Yes | No | No | Yes | 1, 2, 3 | 7, 8, 9, 10 | Yes | Yes | ★★★★★★ |
| Finch et al. | Yes | Yes | No | Yes | 5 | 8, 9, 10 | Yes | Yes | ★★★★★★ |
| Guzzetti et al. | Yes | No | No | Yes | 1, 2, 3, 4 | 6, 7, 8, 10 | Yes | Yes | ★★★★★★ |
| Hosokawa et al. | Yes | Yes | No | Yes | 5 | 6, 7, 9, 10 | Yes | Yes | ★★★★★★ |
| Kokudo et al. | Yes | Yes | No | Yes | 2, 3, 5 | 6, 10 | Yes | Yes | ★★★★★★★ |
| Lalmahomed et al. | Yes | Yes | No | Yes | 1, 2, 3, 4, 5 | 6, 7, 9,10 | Yes | Yes | ★★★★★★★★ |
| Margonis et al. | Yes | Yes | No | Yes | 2, 4, 5 | 6, 7, 9, 10 | Yes | Yes | ★★★★★★★ |
| Matsumura et al. | Yes | No | No | Yes | 1, 2, 3, 4 | 6, 7, 9, 10 | Yes | Yes | ★★★★★★ |
| Memeo et al. | Yes | Yes | No | Yes | 5 | 6, 7, 10 | Yes | Yes | ★★★★★★ |
| Mise et al. | Yes | No | No | Yes | 1, 2, 3, 4 | 6, 7, 10 | Yes | Yes | ★★★★★★ |
| Pandanaboyana et al. | Yes | Yes | No | Yes | 3, 5 | 8, 9, 10 | Yes | Yes | ★★★★★★ |
| Sarpel et al. | Yes | Yes | No | Yes | 4, 5 | 6, 7, 10 | Yes | Yes | ★★★★★★ |
| Spelt et al. | Yes | No | No | Yes | 1, 3, 5 | 7, 9, 10 | Yes | Yes | ★★★★★★ |
| Steward et al. | Yes | Yes | No | Yes | N/A | N/A | Yes | Yes | ★★★★★ |
| Zorzr et al. | Yes | Yes | No | Yes | 2, 5 | 6, 7, 10 | Yes | Yes | ★★★★★★ |
| *: 1=metastasis number, 2=metastasis size, 3=site of tumor (unilateral or bilateral), 4=disease-free interval, 5=diagnosis of metastasis disease (whether synchronous or not), 6=primary site of tumors, 7=primary nodal status, 8=chemotherapy, 9=neoadjuvant chemotherapy, 10=age.  †: Studies fulfilled at least three of the criteria could get one point, studies fulfilled all the criteria could get two point. | | | | | | | | | |

Supplemental table 2. Meta-regression analysis examining the potential modifying role of various study features on OS and RFS.

| **Variables** | **Category or increment** | **OS** | | | **DFS** | | |
| --- | --- | --- | --- | --- | --- | --- | --- |
|  |  | **n** | **Exponentiated coefficient (95%CI)** | **p** | **n** | **Exponentiated coefficient (95%CI)** | **p** |
| Publication year | 1 year increase | 16 | 1.01 (0.99-1.02) | 0.384 | 11 | 0.99 (0.95-1.03) | 0.545 |
| Percentage of males | 1% increase | 13 | 1.01 (0.98-1.04) | 0.413 | 9 | 1.01 (0.98-1.04) | 0.485 |
| Mean/median age | 1 year increase | 12 | 1.01 (0.98-1.05) | 0.468 | 10 | 1.02 (0.99-1.06) | 0.178 |
| Percentage of synchronous metastasis | 1% increase | 11 | 1.00 (0.99-1.01) | 0.818 | 8 | 1.00 (0.99-1.01) | 0.760 |
| Location of primary tumors (percentage of colon cancer) | 1% increase | 9 | 0.99 (0.97-1.01) | 0.233 | 6 | 0.99 (0.97-1.01) | 0.277 |
| Mean/median number of metastases | 1 increase | 6 | 0.98 (0.88-1.09) | 0.612 | 7 | 0.99 (0.91-1.07) | 0.680 |
| Mean/median size of largest metastases | 0.1 cm increase | 11 | 0.95 (0.85-1.05) | 0.263 | 9 | 0.98 (0.89-1.09) | 0.691 |
| Percentage of negative margin (total) | 1% increase | 14 | 1.00 (0.99-1.01) | 0.269 | 10 | 1.00 (0.99-1.01) | 0.757 |
| Mean/median follow-up period | 1 month increase | 10 | 1.01 (0.99-1.03) | 0.415 | 8 | 1.02 (0.99-1.04) | 0.177 |
| Percentage of neoadjuvant chemotherapy (total)^†^ | 1% increase | 9 | 1.00 (0.99-1.00) | 0.975 | 8 | 1.00 (0.99-1.00) | 0.994 |
| Quality scores | 1 increase | 15 | 0.96 (0.81-1.13) | 0.603 | 10 | 0.92 (0.77-1.11) | 0.346 |

*OS: overall survival, RFS: recurrence-free survival, CI: confidence interval, n: number of study arms.
